# Omega-9 Oleic Acid Induces Fatty Acid Oxidation and Decreases Organ Dysfunction and Mortality in Experimental Sepsis

**DOI:** 10.1371/journal.pone.0153607

**Published:** 2016-04-14

**Authors:** Cassiano Felippe Gonçalves-de-Albuquerque, Isabel Matos Medeiros-de-Moraes, Flora Magno de Jesus Oliveira, Patrícia Burth, Patrícia Torres Bozza, Mauro Velho Castro Faria, Adriana Ribeiro Silva, Hugo Caire de Castro-Faria-Neto

**Affiliations:** 1 Laboratório de Imunofarmacologia, Instituto Oswaldo Cruz, FIOCRUZ, 21040–900 Rio de Janeiro, RJ, Brazil; 2 Departamento de Biologia Celular e Molecular, Instituto de Biologia, Universidade Federal Fluminense, 24020–15 Niterói, RJ, Brazil; 3 Departamento de Medicina Interna, Faculdade de Ciências Médicas, Universidade do Estado do Rio de Janeiro, 20550–900 Rio de Janeiro, RJ, Brazil; 4 Universidade Estácio de Sá, Programa de Produtividade Científica, Rio de Janeiro, RJ, Brazil; University of Pecs Medical School, HUNGARY

## Abstract

Sepsis is characterized by inflammatory and metabolic alterations, which lead to massive cytokine production, oxidative stress and organ dysfunction. In severe systemic inflammatory response syndrome, plasma non-esterified fatty acids (NEFA) are increased. Several NEFA are deleterious to cells, activate Toll-like receptors and inhibit Na^+^/K^+^-ATPase, causing lung injury. A Mediterranean diet rich in olive oil is beneficial. The main component of olive oil is omega-9 oleic acid (OA), a monounsaturated fatty acid (MUFA). We analyzed the effect of OA supplementation on sepsis. OA ameliorated clinical symptoms, increased the survival rate, prevented liver and kidney injury and decreased NEFA plasma levels in mice subjected to cecal ligation and puncture (CLP). OA did not alter food intake and weight gain but diminished reactive oxygen species (ROS) production and NEFA plasma levels. Carnitine palmitoyltransferase IA (CPT1A) mRNA levels were increased, while uncoupling protein 2 (UCP2) liver expression was enhanced in mice treated with OA. OA also inhibited the decrease in 5' AMP-activated protein kinase (AMPK) expression and increased the enzyme expression in the liver of OA-treated mice compared to septic animals. We showed that OA pretreatment decreased NEFA concentration and increased CPT1A and UCP2 and AMPK levels, decreasing ROS production. We suggest that OA has a beneficial role in sepsis by decreasing metabolic dysfunction, supporting the benefits of diets high in monounsaturated fatty acids (MUFA).

## Introduction

Sepsis is a medical condition caused by severe infection that involves systemic inflammation [1] and metabolic changes [2], resulting in multiple organ failure (MOF) and high morbidity and mortality [3].

Metabolic dysfunctions during sepsis include alterations in lipid metabolism and decreased fatty acid oxidation [4]. Consequently, plasma free fatty acids increase, resulting in increased tissue lipolysis in organs such as the liver, kidney, heart and skeletal muscle. This results in harmful consequences for patients with diverse pathological conditions [2, 4–7].

Under physiological conditions, approximately 0.1 to 2 mol of fatty acids are in complex with albumin [8]. As albumin is synthesized in the liver, patients with liver dysfunction, such as cirrhosis and sepsis, have lower levels of albumin, which favors increased serum free fatty acid [9]. The non-esterified fatty acids (NEFA) bind to fatty acid-binding protein (FABP) and acyl-CoA (acyl-CoA-binding protein), transporting them to either the mitochondria and peroxisomes, where they are oxidized, or to the nucleus, where they activate gene transcription [10]. These processes are tightly enzymatically controlled. The entry of fatty acids into the mitochondrial matrix is regulated by the enzyme carnitine palmitoyltransferase I [11], a rate-limiting step of fatty acid β-oxidation. The enzyme uncoupling protein-2 (UCP2) is also associated with increased fatty acid oxidation [12] and NEFA reduction [13]. The 5' AMP-activated protein kinase (AMPK) plays a crucial role in energy homeostasis by increasing catabolic pathways, including β-oxidation, or blocking anabolic processes, such as fatty acid synthesis [14]. Decreased hepatic expression of fatty acid-metabolizing enzymes occurs during sepsis and endotoxemia [15] and could account for the increased plasma NEFA concentrations in septic patients.

Polyunsaturated fatty acids (PUFA) modulate immune system functions. For example, n-3 PUFA decreases the severity of inflammatory disorders [16], but less attention has been paid to the effects of MUFA on the immune system [17]. Olive oil is the primary source of fat in the Mediterranean diet and has oleic acid (OA) [18] as the primary component (a MUFA of the omega-9 family). The Mediterranean diet reduces cardiovascular disease, the incidence of Parkinson's and Alzheimer's disease and cancer [19]. This diet also results in downregulation of circulating inflammatory biomarkers [20] and oxidative stress. Rats treated with extra virgin olive oil had reduced levels of malondialdehyde induced by herbicide exposure and showed reduced hepatic oxidative stress [21]. Previous reports have indicated that MUFA might represent a useful tool in the design of dietary regimens for obesity, cardiovascular diseases [22] and type 2 diabetes [23]. Considering the beneficial effects of MUFA, we aimed to study the effect of OA on lipid metabolism during sepsis. Here, we evaluated the plasma NEFA levels and expression of liver enzymes directly involved in fatty acid oxidation as well as the impact of these modifications on sepsis outcome.

## Methods

### Animals

We used male Swiss-Webster (SW) mice (25 to 30 g) from the Oswaldo Cruz Foundation breeding unit, Rio de Janeiro, Brazil. The animals were kept at 22°C with a 12 hour light/dark cycle and free access to food and water. The animals were weighed on days 1, 7 and 14, and the food intake was estimated per body weight for each cage.

Animals used in our experiments were sacrificed for liver removal and were euthanized due to sepsis complications, such as shock and multiple organ failure. We determined whether daily pretreatment with OA for 14 days would be protective against sepsis complications and improve the survival of septic animals. We used humane endpoints in the experiments, using a lethal anesthesia before the animals were moribund to analyze molecular parameters 24 hours after sepsis induction. We euthanized animals prior to the end of our experiment with a lethal dose of ketamine and xylazine, following the guidelines of our Institutional Animal Ethics Committee.

We described the animal deaths without euthanasia in the study protocol submitted to our Institutional Animal Ethics Committee. Our ethics committee specifically reviewed and approved the mortality aspects of the protocol. It was a specific aim of the study to analyze whether oleic acid would modulate lipid metabolism and have protective effects against organ failure and shock, improving survival during experimental sepsis. We could not use pain relievers because it has already been shown that pain relievers can also modulate lipid metabolism, and they may have protective effects, potentially biasing results. Pain relievers, such as nonsteroidal anti-inflammatory drugs, inhibit the production of the lipid mediator thromboxane B2, for example, which causes platelet aggregation and leads to the synthesis of lipoxin A4, accelerating resolution and affecting the sepsis and endotoxemia outcome [24–26]. Thirteen animals died in the CLP group, and 8 animals died in the oleic acid-treated septic group. Surviving animals were healthy and behaved normally. We monitored the health of the animals three times a day. We avoided any animal suffering and distress using anesthesia with ketamine and xylazine during surgical procedures, and all efforts were made to minimize suffering.

This study was carried out in strict accordance with the recommendations in the Guide for the Care and Use of Laboratory Animals of the Animal Welfare Committee of the Oswaldo Cruz Foundation.

### Ethics statement

The Animal Welfare Committee of the Oswaldo Cruz Foundation under license number LW-36/10 (CEUA/FIOCRUZ) approved the experiments in these studies. The same institution that provided ethical approval created the guidelines followed by this Committee.

### Oleic acid administration

Mice were administered daily doses of OA (Sigma) for 14 days. Oleate solution was prepared by addition of water. NaOH was slowly added until the pH reached 12.0. This mixture was sonicated, and after complete oleate solubilization, the pH was carefully adjusted to 7.6 with diluted HCl. Each animal received 0.28 mg of OA (100 μL) per day by gavage. Control mice received 100 μL of saline orally per day.

### Cecal ligation and puncture (CLP)

Mice orally received OA or saline for 14 days. On the 15^th^ day, polymicrobial sepsis was induced by CLP performed as described in Araujo et al. (2012) with minor modifications [27]. Briefly, mice were anesthetized with intraperitoneal injections of ketamine (100 mg/kg) (Cristália) and xylazine (10 mg/kg) (Syntec). After aseptic procedures with 70% ethanol, an incision was made through the linea alba. The cecum was exposed, ligated with sterile 3–0 silk and perforated twice with an 18 gauge needle. A small amount of fecal material was extruded through the wounds, and the cecum was gently pushed into the abdomen. The area was sutured with nylon 3–0 (Shalon) in two layers. All mice received volemic reposition of 1 mL of sterile saline subcutaneously. Six hours after surgery, mice received the antibiotic imipenem (10 mg/kg) intraperitoneally in the opposite site from the surgery. Sham mice were subjected to the same procedures described above, but the cecum was neither ligated nor punctured.

### Clinical score

Six and 24 hours after surgery, all mice had their clinical score evaluated. Time points for analysis were chosen based on previous data [27]. The score consisted of analyzing the following parameters: presence of piloerection, altered respiration rate, fecal alteration, lacrimation/eyelid changes, contraction of the abdomen, lack of strength when grasping, change in body temperature, alert response (scape after touch), exploration of the environment and compromised locomotor activity. For every parameter present, we gave 1 point, and in the absence of the parameter analyzed, no points were given. Then, the points were computed for each mouse. A score of 0 indicated that the mouse did not present any clinical alteration, a score between 1 and 3 indicated mild sepsis, between 4 and 7 indicated moderate sepsis and between 8 and 10 indicated severe sepsis [27].

### Biochemical analysis

Mice were fasted for 12 hours with water *ad libitum*, and blood was then collected by cardiac puncture. Serum was separated by centrifugation and used for the quantification of albumin, creatinine and oxaloacetic transaminase. The quantifications were made using the dry chemistry methodology with a Vitros 250 (Ortho Clinical—Johnson & Johnson).

### Plasma non-esterified fatty acid (NEFA) quantification

Plasma concentrations of the predominant NEFA—palmitic, oleic, linoleic, palmitoleic, and stearic acids—were determined by high-performance liquid chromatography (HPLC) as described by Puttman et al. [28]. Methodological details were described in a previous publication [5].

### Thiobarbituric acid reactive species (TBARS)

TBARS were measured in whole livers as previously described [29] with minor modifications. Briefly, 300 μg of protein was mixed with an equal volume of 0.67% thiobarbituric acid (Sigma Chemical, USA) and then heated at 96°C for 30 min. TBARS were determined by the absorbance at 535 nm. The results were expressed as malondialdehyde (MDA, ɛ = 1.56 x10^5^ M^-1^ cm^-1^) in 300 μg of protein.

### Western blot analysis

Detection of UCP2 was performed by western blotting. Briefly, organs were perfused with 20 mM ethylenediaminetetraacetic acid (EDTA, pH 7.4). Liver tissues cut into small pieces were homogenized at 4°C in lysis buffer containing protease inhibitors (Roche, AG, Basel, Switzerland). Periepididymal adipose tissues were homogenized at 4°C in RIPA buffer with protease inhibitors (Roche, AG, Basel, Switzerland) and phosphatase inhibitor cocktails (Roche). Tissues were stored at -20°C for further protein quantification. Western blot analyses were performed with whole liver and adipose tissues lysates (40 μg of proteins) using anti-UCP2 (1:1000 dilution, Abcam) and anti-β-actin (1:15000 dilution, Sigma). Detection was performed with the Super Signal Chemiluminescence kit (Pierce), exposing the membrane to an autoradiograph film (GE Healthcare). Bands were digitalized and analyzed by size and intensity by the Image Master 2D Elite program. Anti-AMPK (Cell Signaling) was used, and infrared-labeled goat anti-mouse IRDye 800CW secondary antibodies (Li-Cor Biosciences) were added to bind to the primary antibody. Detection was performed with an Odyssey scanner. Bands were digitalized and analyzed by size and intensity by the Image Studio 3.1 [30].

### Isolation of RNA and reverse transcription-polymerase chain reaction (RT-PCR) analysis

Total RNA was extracted from perfused whole liver using TRIzol reagent (Life Technologies, CA, USA) according to the manufacturer’s instructions. First-strand cDNA synthesis was performed using total RNA primed with oligo (dT) and SuperScript II RT (SuperScript First-Strand System for RT-PCR, Invitrogen), following the manufacturer’s recommendations. The PCR protocol for CPT1A consisted of 40 cycles of 90°C for 1 min, 60°C for 1 min and 72°C for 1 min. The PCR protocol for GAPDH consisted of 35 cycles of 94°C for 1 min, 57°C for 1 min and 72°C for 1 min. PCR was performed using CPT1A primers GAACTTGCCCATGTCCTTGT (right) and CCAGGCTACAGTGGGACATT (left) and GAPDH primers ATACCAGGAA ATGAGCTTGACAAAGT (right) and CCAGGTTGTCTCCTGGGACT (left) (GenBank). The PCR products were visualized on 6% polyacrylamide gel using silver staining. Images were analyzed using the ImageMaster 2D Elite program.

### Statistical analysis

Results were analyzed with a one-way ANOVA followed by Newman-Keuls tests or Student’s t test with GraphPad Prism 5.0. Values with p < 0.05 were considered significant. Mortality curves were analyzed by the log-rank test. All data are presented as the mean ± SEM.

## Results

### Oleic acid administration ameliorated clinical score in septic Swiss mice

**Fig 1:**
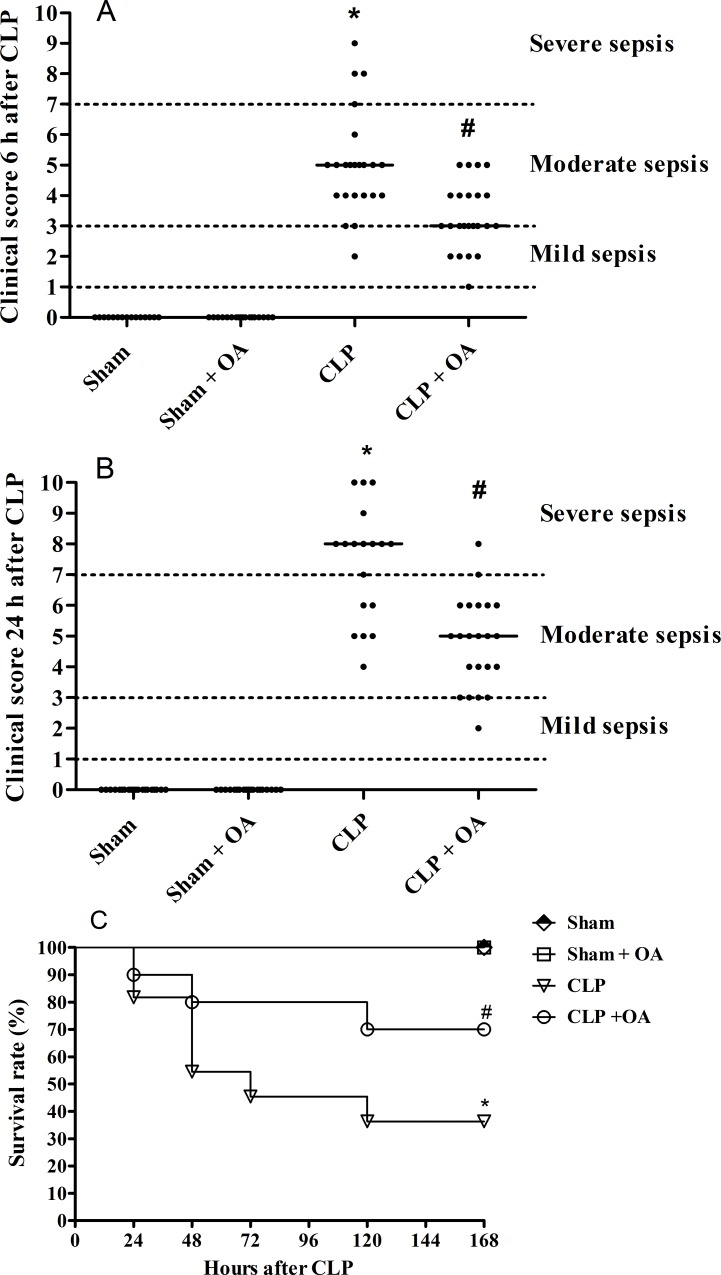
Oleic acid administration improves survival and ameliorates clinical score in septic mice. Animals were treated with oleic acid for 14 days. On the 15^th^ day, mice were subjected to CLP, and (A) 6 and (B) 24 hours after surgery, the clinical score was evaluated as described in the Materials and Methods. (C) The survival rate was assessed for 7 days after CLP. Control groups received saline. Each group consisted of 15 to 23 animals (clinical score at 6 hours: sham = 15 animals, sham + OA = 18 animals and CLP and CLP + OA = 23 animal; at 24 hours: sham and sham + OA = 18 animals, CLP = 18 animals, CLP + OA = 22 animals) in 3 independent experiments. For survival rates, 10 animals from each group were analyzed. This is a representative curve from 3 independent experiments. p < 0.05 * CLP vs sham, # CLP vs CLP plus oleic acid; log-rank test for mortality and one-way ANOVA followed by Newman-Keuls test for the clinical score.

OA administration prevented several alterations after sepsis induction. Therefore, we investigated the effect of OA on sepsis severity and mortality using clinical scores [27, 29] (as described in Materials and Methods). Six hours after CLP, mice presented clear clinical signs of sepsis (Fig 1A), and at 24 hours, most animals in the sepsis group had severe sepsis (Fig 1B). OA pretreatment reduced the clinical score 6 and 24 hours after CLP (Fig 1A and 1B) and improved the clinical outcome, as reflected in an increased survival rate of OA treated-animals (Fig 1C).

### Effect of OA on caloric balance and food intake

**Fig 2:**
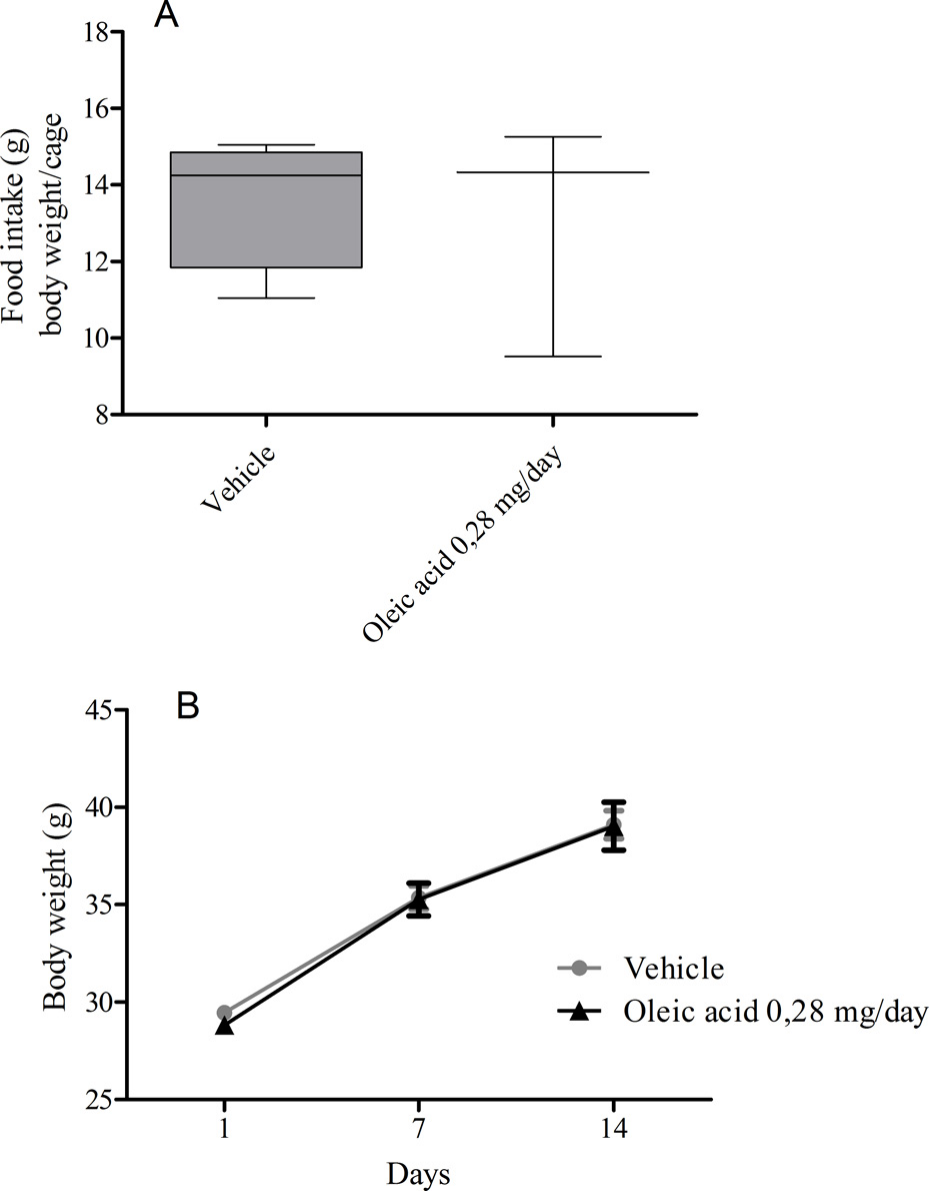
Oleic acid administration does not affect food intake and body weight of mice. (A) After 14 days of oleic acid administration, we estimated the food intake per body weight per cage. (B) Animals were followed for 14 days after OA administration and weighed on days 1, 7 and 14. Controls received the same volume of saline. Values represent the mean *±* SEM from at least 13 animals.

Because we provided caloric supplementation to the animals, i.e., daily doses of a fatty acid for 14 days, we measured the weight gain during this treatment. Animals that received vehicle or OA were weighed on the 1st, 7th and 14th days. This treatment did not affect weight gain or food intake during the 14 days of treatment (Fig 2).

### OA decreased renal and hepatic dysfunction in mice subjected to CLP

**Fig 3:**
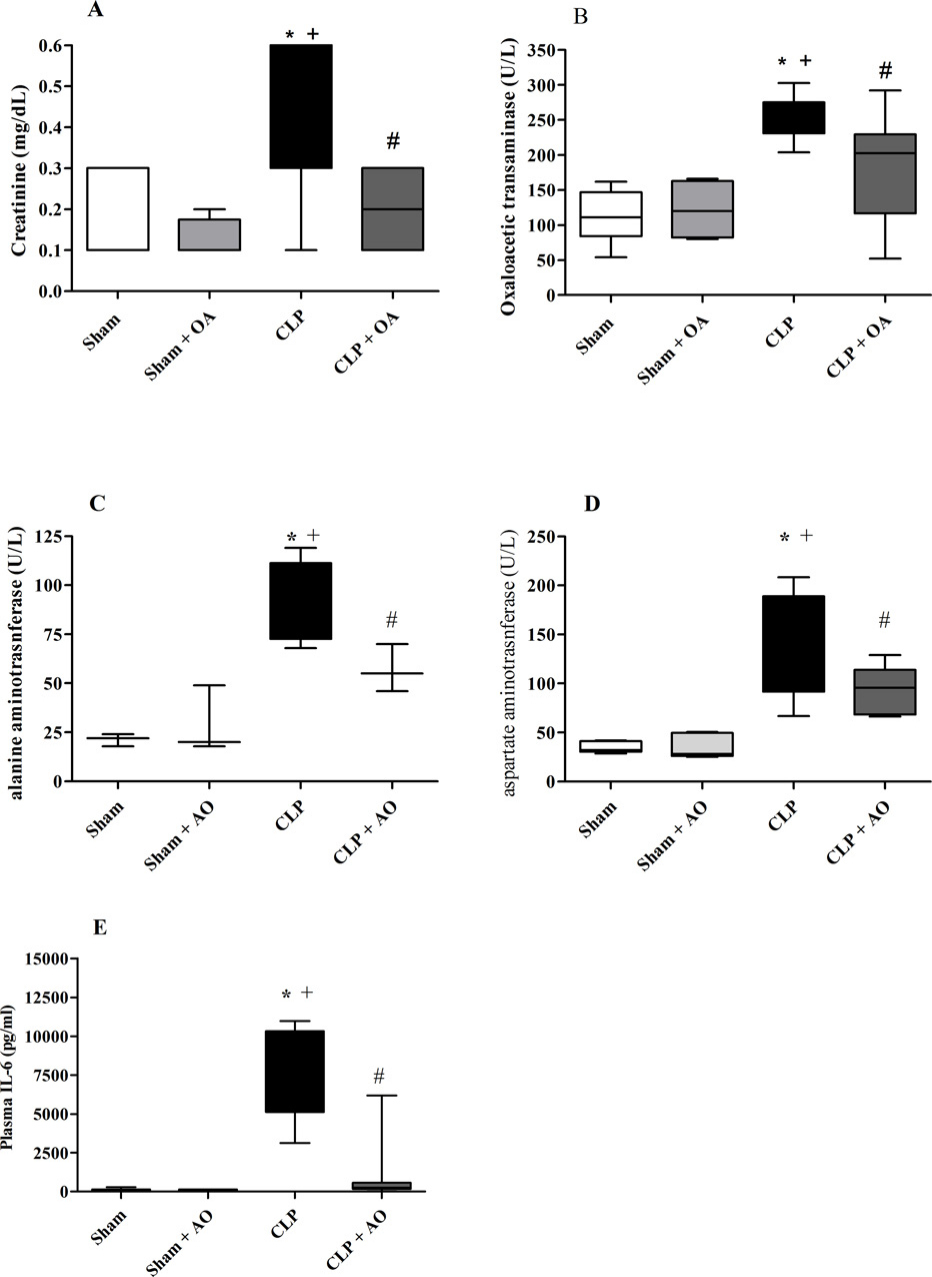
Oleic acid treatment ameliorates renal and hepatic functions in mice subjected to CLP. Animals were treated with oleic acid for 14 days. On the 15^th^ day, mice were subjected to CLP, and 24 hours after surgery, serum was collected for the quantification of (A) creatinine, (B) glutamic-oxaloacetic aminotransferase and (C) alanine aminotransferase. Each bar represents the mean *±* SEM from 5–13 animals per group. p < 0.05 * CLP vs sham, + CLP vs sham plus OA, # CLP vs CLP plus oleic acid.

Twenty-four hours after CLP, septic mice showed increased plasma creatinine levels (Fig 3A) and hepatic transaminases (Fig 3B and 3C), which are markers of kidney and hepatic dysfunction. OA administration decreased creatinine, glutamic-oxaloacetic transaminase and alanine aminotransferase levels in septic mice (Fig 3).

### OA treatment reduced plasma NEFA concentrations in septic animals

**Fig 4:**
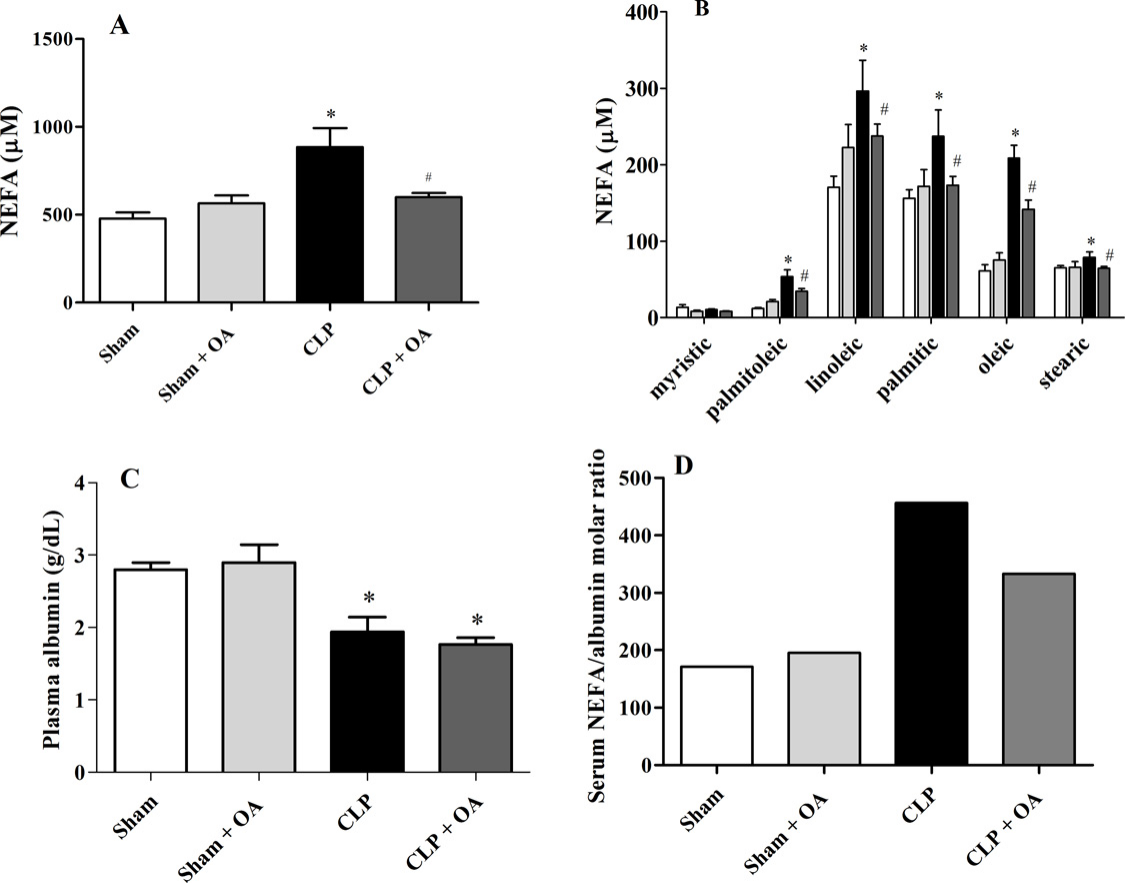
Plasma NEFA concentrations are reduced after oleic acid treatment in septic animals. Mice were treated for 14 days with oleic acid. On the 15^th^ day, mice were subjected to CLP. Twenty-four hours after CLP, blood was collected for albumin quantification. (A)Total NEFA concentration (sum of average concentrations of the five NEFA) 24 hours after CLP in OA-treated and untreated animals. (B) Plasma concentrations of palmitoleic, linoleic, palmitic, oleic and stearic acids. (C) Plasma albumin levels. (D) Ratio of serum NEFA and albumin. Values represent the mean *±* SEM of at least 5 animals. (Total NEFA and single fatty acid: sham = 7 animals, sham + OA = 5 animals, CLP = 6 animals and CLP + OA = 8 animals; Albumin and ratio of serum NEFA and albumin: sham = 5 animals, sham + OA = 6 animals, CLP = 5 animals and CLP + OA = 6 animals). The results are representative of 3 independent experiments. p < 0.05 * CLP vs sham, # CLP vs CLP plus oleic acid.

Plasma NEFA levels frequently increase in pathological conditions [2, 15, 31], and they may have a deleterious role in septic patients [2, 32]. Accordingly, we observed that septic mice had increased levels of total plasma NEFA (Fig 4A), including palmitoleic acid (monounsaturated, omega-7), linolenic acid (polyunsaturated, ω-6), palmitic acid (saturated), stearic acid (saturated) and OA (monounsaturated, ω-9), representing approximately 90% of plasma NEFA [33] (Fig 4B). In our previous work, we reported that OA decreased NEFA in healthy mice [34], and this was also found when OA was administered to septic mice (Fig 4A and 4B). Albumin is the main fatty acid transporter in the plasma, and septic patients have been shown to have lower albumin levels [8]. Animals subjected to CLP had lower levels of albumin, and OA treatment did not alter the levels of plasma albumin (Fig 4C). As the ratio of NEFA/albumin is important for the prognosis of the disease [9], we showed that OA treatment decreased the ratio, which is associated with a favorable prognosis (Fig 4D).

### Oleic acid increased the transcription of the CPT1A gene

**Fig 5:**
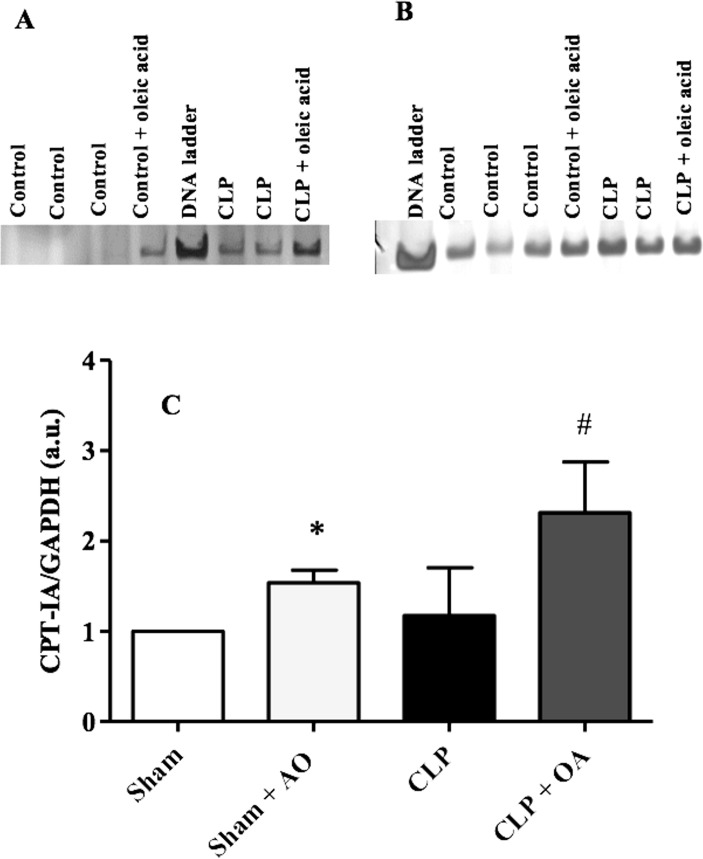
Oleic acid increases the transcription of the CPT1A gene in septic mice. Swiss mice were treated for 14 days with oleic acid. On the 15^th^ day, mice were subjected to CLP. The liver was removed 24 hours after CLP. CPT1A mRNA was detected by RT-PCR. A representative gel of (A) CPT1A and of the (B) control GAPDH gene transcription. The loading control was GAPDH. (C)The bands were analyzed by densitometry and are represented as the CPT1A/GAPDH ratio. Values represent mean and SEM from 5–6 animals per group.

Unsaturated fatty acids are endogenous ligands for the peroxisome proliferator-activated receptor (PPAR) [35]. OA binds to PPAR [36]. Because PPARα is highly expressed in the liver, we assessed PPARα activation by measuring the liver expression of the PPARα-regulated gene carnitine palmitoyltransferase (CPT1A). OA administration increased CPT1A mRNA levels in control mice. OA effect in CPT1A enhancement was more evident in mice subjected to CLP (Fig 5).

### Oleic acid treatment decreased MDA production, induced UCP2 and AMPK in septic mice

**Fig 6:**
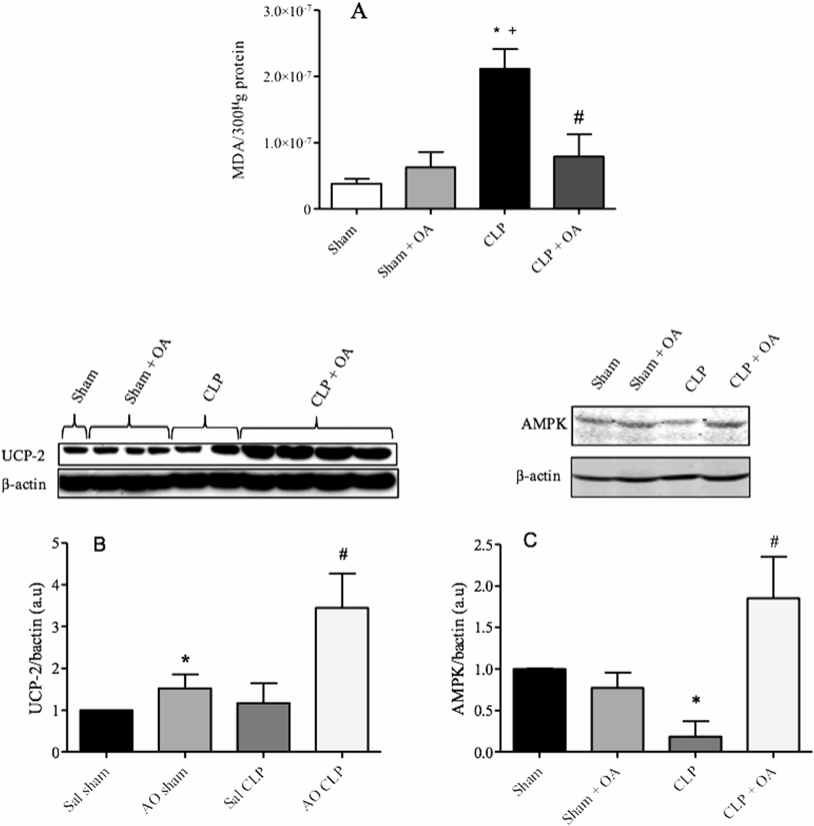
Oleic acid treatment decreases MDA formation, and induces UCP2 and AMPK in septic mice. Mice were treated for 14 days with oleic acid, and on the 15^th^ day, CLP was performed. The liver was removed 24 hours after CLP for analysis of (A) MDA and (B) UCP2 by western blotting. The graph shows the densitometric analysis of the UCP2, AMPK and β-actin bands, as described in the methods. The results are expressed as the mean *±* SEM of 5–6 animals. (* and +) p < 0.05 compared to sham and sham + OA (respectively) and (#) compared to CLP.

Production of reactive oxygen species (ROS) by phagocytic cells (as neutrophils), endothelial cells and hepatocytes is increased during sepsis. This overexpression may be deleterious to the host [37, 38]. OA decreased ROS production in septic mice, as determined by TBARS quantification in the liver (Fig 6A). UCP2 is a mitochondrial protein responsible for the reduction in ROS production [39, 40]. OA treatment increased UCP2 expression to levels (Fig 6B). AMPK is a key enzyme that controls lipid metabolism, including lipid oxidation and lipid synthesis [41]. AMPK activation increases lipid oxidation through PPARα and UCP2 [42]. Here, OA was shown to increase the levels of AMPK (Fig 6C).

## Discussion

Despite advances in care of septic patients, incidence and mortality remain high [3, 43]. The lack of treatments that effectively reduce the excessive response of the host to infections is thought to be one of the foremost obstacles to reducing sepsis mortality [44].

Different diets can affect the host immune response [45]. The Mediterranean diet has been associated with increased longevity, improved overall health, and reduced incidence and mortality of cancer and of other chronic diseases. In the Mediterranean diet, the primary source of fat is olive oil, which is mainly composed of OA. An olive oil-enriched diet protected against endotoxic shock in isogenic C57Bl/6J mice [46]. Our results showed that pretreatment with OA prevented the deterioration in clinical status of septic animals and increased the survival rate. Consistent with our data, the administration of immunomodulatory diets containing fish oil (rich in omega-3) increased survival and reduced hospitalization time of ICU patients with septic shock or ARDS [47].

Morbidity and death in septic patients are associated with liver and respiratory failure [48, 49]. Indeed, acute renal failure in ICU patients was observed during sepsis [50, 51]. In our work, OA was able to mitigate the severity of the disease, decreasing the clinical score and also preventing renal and hepatic dysfunction. We found increases in different biochemical markers of liver injury, but due to strain specificity, these levels may be lower than those found in other reports. Although we believe they are important markers in the improvement of animal survival, we cannot assume they are the sole cause of the improved survival. Our study has several limitations due to the model of sepsis induction used and its clinical relevance. The CLP model is one of the most commonly used models to study sepsis due to the similarity to human sepsis. Nevertheless, there are many different ways to perform CLP, and this causes variability in the sepsis results [52]. These differences include levels of organ injury markers, susceptibility to organ injury, and death, and they can be related to the CLP procedure itself due to the use of antibiotics and posology, number of cecal punctures, needle size, or strain, gender and other factors [52, 53]. According to our data, nitro-oleic acid-treated animals challenged with LPS had less severe multiple organ dysfunctions than animals receiving only LPS [54]. OA affects several different biological processes, but its detailed mechanism of action is not completely understood.

During sepsis, a decrease in fatty acid oxidation occurs, causing an increase in NEFA in plasma and a reduction in energy supply to the organs [2, 55]. Furthermore, patients with sepsis and leptospirosis have high levels of NEFA in the blood [5, 56], which are associated with hypoalbuminemia and liver failure. Thus, the decrease in energy supply to the organs contributes to multiple organ failure and death [2].

We showed that septic animals had a significant increase in NEFA and that OA treatment reduced NEFA levels. Similarly, in our previous report we showed that a single dose of OA administered intravenously or orally reduced plasma NEFA in uninfected Swiss mice [34]. Supplementation with long-chain MUFA decreased plasma free fatty acids in obese mice [57]. We then investigated the expression of proteins involved in fatty acid oxidation because increased fatty acid oxidation reduces plasma NEFA. PPAR is a transcription factor belonging to the nuclear receptor family and acts as lipid sensor, interpreting fatty acids signals linking lipid metabolism and inflammation [58, 59]. OA binds to all three PPAR isoforms [36]. A potent PPARα activator derived from tomato juice, 13-oxo-9,11-octadecadienoic acid, decreased plasma and hepatic triglycerides in obese diabetic mice [60]. Furthermore, PPAR activation is important in preventing LPS-induced acute liver damage by regulating oxidative/nitrosative stress and STAT1 inflammatory signaling pathways [61].

Many genes controlled by PPARα, including CPT1A and FABP1, are involved in lipid metabolism in humans and mice; therefore, PPARα activation has a major impact on gene regulation in hepatocytes [62]. For entry into the mitochondria to undergo oxidation, fatty acids must be processed by CPT1A. Therefore, this enzyme is an important step in the regulation of fatty acid oxidation [11, 63]. It was also reported that LPS-challenged mice showed an increase in NEFA and a reduction in the expression of CPT1A [2], leading to lipid accumulation in the body [64]. We showed that OA treatment increased CPT1A mRNA expression in the liver.

Fatty acids activate AMPK and increase CPT1A activity, thus increasing lipid oxidation [65]. AMPK is an essential molecular player in energy homeostasis at both the cellular and whole-body levels [66]. Activated AMPK induces fatty acid uptake and oxidation in muscle cells, blocks fatty acid synthesis in the liver and decreases lipolysis in adipose tissue [66]. NEFA induce AMPK phosphorylation in the liver, increasing the transcriptional activity of PPARα. Our data showed that septic animals treated with OA increased the expression of AMPK, which could participate in the induction of liver CPT1A mRNA. Because CPT1A expression is controlled by PPARα [67], and OA is a ligand of PPARα [36] that activates AMPK, we suggest that, at least in part, the effect of OA on CPT1A expression involves AMPK/PPAR activation. Consistent with our data, a report showed that obese diabetic mice supplemented with long-chain MUFA had increased mRNA expression of CPT1A [57]. We hypothesize that in our model, OA induced an increase in CPT1A, consequently increasing plasma fatty acid oxidation and reducing NEFA in the plasma.

Increased oxidative stress also occurs during sepsis and results in overproduction of ROS, cell damage, multiple organ failure and death [38, 68]. OA prevented excessive production of ROS during sepsis. Mice receiving an olive oil-rich diet had lower levels of MDA in a model of oxidative stress caused by injection of acetaminophen [69]. Upregulation of UCP2 has been suggested to be a protective mechanism against excessive lipid exposure and the associated increase in ROS production, lending further support to the role of UCP2 as an antioxidative agent [70]. UCP (thermogenin) is located in the inner membrane of the mitochondria. Its primary function is to translocate protons from the intermembrane space to the matrix of the mitochondria [71]. A pharmacologically induced decrease in plasma NEFA was accompanied by increased activation of PPAR and UCP2 expression in the liver [72]. We showed that OA increased UCP2 protein levels. Thus, the decrease in ROS production by OA may be, at least in part, related to its ability to control UCP expression. Supporting our hypothesis, PPARα knockout animals had increased MDA production compared to wild-type animals [61]. Additionally, OA increased expression of UCP2 in a PPARα-dependent pathway in rat hepatocytes, and the PPARα agonist Wy-14643 stimulated UCP2 mRNA levels [73]. UCP2 is associated with increased oxidation of fatty acids [12] and a consequent reduction in NEFA production [13]. Hence, we suggest OA enhance UCP2, reducing ROS production and increasing fatty acid oxidation, thus lowering plasma NEFA during experimental sepsis [42, 74].

**Fig 7:**
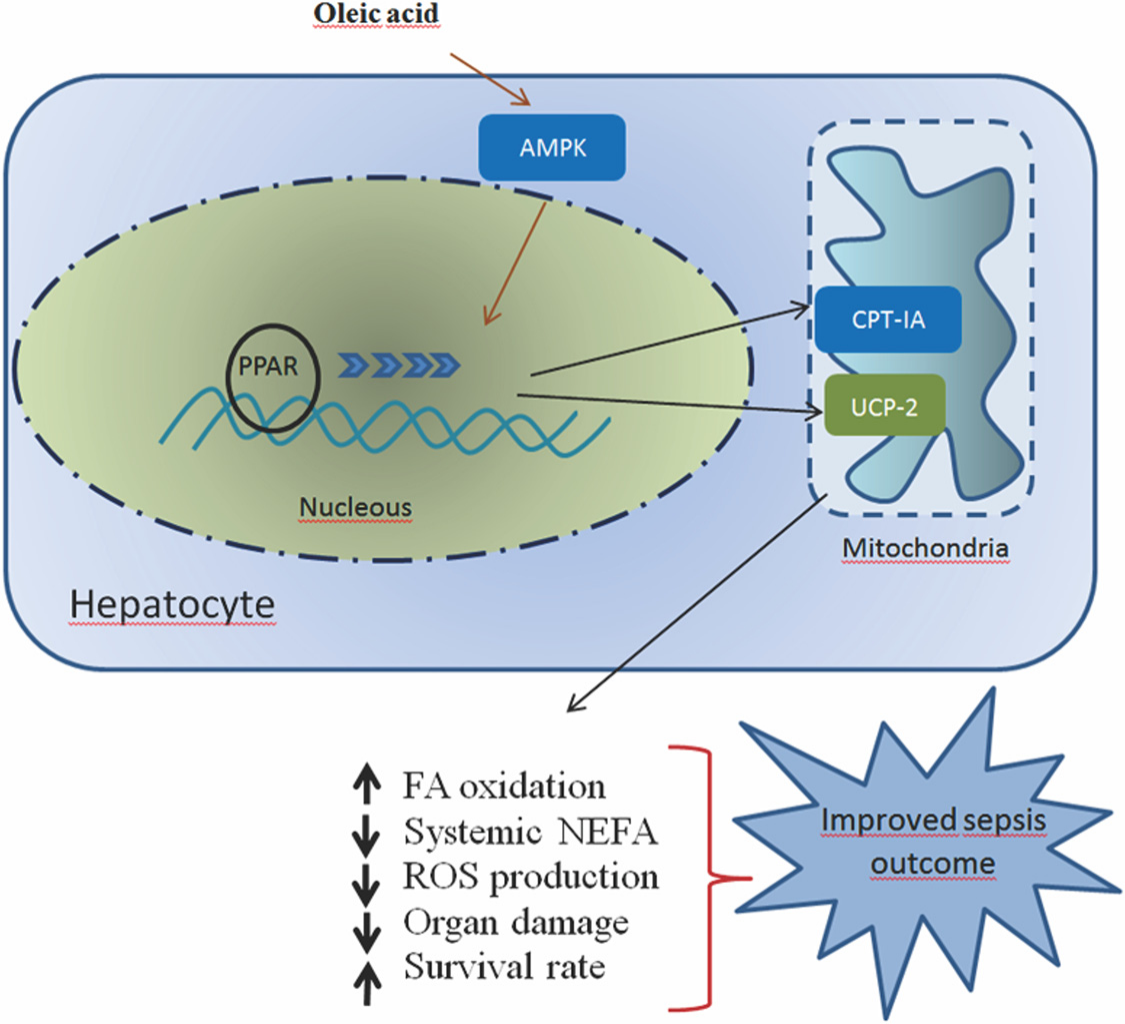
OA alters fatty acid metabolism and organ dysfunction and improves sepsis outcome. CPT1A –carnitine palmitoyltransferase 1A; PPAR–peroxisome proliferator-activated receptor, UCP2 –uncoupling protein 2; AMPK—5' AMP-activated protein kinase. OA activates AMPK, increases CPT1A and UCP2 and decreases fatty acid synthesis, resulting in an increase in oxidative processes. PPAR activation leads to an increase in the expression of CPT1A and UCP2; consequently, the fatty acid oxidation will be enhanced. Augmented fatty acid oxidation leads to a decrease in the NEFA plasma levels. Decreased NEFA and ROS levels would improve organ dysfunction and increase survival rate.

In summary, treatment with OA caused substantial changes in the metabolism of septic mice and showed significant protective effects. Pretreatment with OA prevented both clinical impairment and organ damage and increased the survival rate. Importantly, OA treatment affected neither the animal weight gain nor food intake. OA treatment induced genes involved in fatty acid oxidation and subsequent fatty acid degradation. NEFA reduction was followed by an increase in the expression of two essential proteins in the oxidation of fatty acids, CPT1A and UCP2 (Fig 7). Consistent with other studies recommending a high MUFA diet in type 2 diabetes [23] and in the composition of dietary regimens for obesity and cardiovascular disease [22], we show that high MUFA diets appear to be effective in reducing NEFA during infection and should be recommended in dietary regimens to prevent and/or ameliorate infectious diseases.
